# Genetic interaction of *DISC1* and *Neurexin* in the development of fruit fly glutamatergic synapses

**DOI:** 10.1038/s41537-017-0040-6

**Published:** 2017-10-27

**Authors:** Himani Pandey, Katia Bourahmoune, Takato Honda, Ken Honjo, Kazuki Kurita, Tomohito Sato, Akira Sawa, Katsuo Furukubo-Tokunaga

**Affiliations:** 1 Life and Environmental Sciences, University of Tsukuba, Tsukuba, 305-8572 Japan; 2 Department of Psychiatry, Johns Hopkins University School of Medicine, Baltimore, MD USA

## Abstract

Originally identified at the breakpoint of a (1;11)(q42.1; q14.3) chromosomal translocation in a Scottish family with a wide range of mental disorders, the *DISC1* gene has been a focus of intensive investigations as an entry point to study the molecular mechanisms of diverse mental dysfunctions. Perturbations of the *DISC1* functions lead to behavioral changes in animal models, which are relevant to psychiatric conditions in patients. In this work, we have expressed the human *DISC1* gene in the fruit fly (*Drosophila melanogaster*) and performed a genetic screening for the mutations of psychiatric risk genes that cause modifications of *DISC1* synaptic phenotypes at the neuromuscular junction. We found that *DISC1* interacts with *dnrx1*, the *Drosophila* homolog of the human *Neurexin* (*NRXN1*) gene, in the development of glutamatergic synapses. While overexpression of *DISC1* suppressed the total bouton area on the target muscles and stimulated active zone density in wild-type background, a partial reduction of the *dnrx1* activity negated the *DISC1*–mediated synaptic alterations. Likewise, overexpression of *DISC1* stimulated the expression of a glutamate receptor component, DGLURIIA, in wild-type background but not in the *dnrx1* heterozygous background. In addition, *DISC1* caused mislocalization of Discs large, the *Drosophila* PSD-95 homolog, in the *dnrx1* heterozygous background. Analyses with a series of domain deletions have revealed the importance of axonal localization of the DISC1 protein for efficient suppression of DNRX1 in synaptic boutons. These results thus suggest an intriguing converging mechanism controlled by the interaction of *DISC1* and *Neurexin* in the developing glutamatergic synapses.

## Genetic risk factors: Mapping interaction in fruit flies

Fruit fly models uncover a potential new mechanism by which two schizophrenia risk factor genes interact to alter synaptic junctions. DISC1 gene alterations have previously been linked to psychiatric anomalies, although the gene has not been formally recognized as a schizophrenia risk factor. A US-Japan research collaboration led by the University of Tsukuba’s Katsuo Furukubo-Tokunaga expressed human DISC1 in fruit fly synapses to better understand the changes that take place when gene disruption leads to overexpression. The team found that overexpression of DISC1 affected the expression of the fruit fly counterpart to human ‘neurexin,’ a known risk factor for conditions including schizophrenia and autism spectrum disorders. The interaction between neurexin and DISC1 also influenced other synapse-altering genes. Further research is warranted to explore the roles of DISC1 and neurexin in psychiatric disease.

## Introduction

Since the discovery in a Scottish family with a (1;11)(q42.1; q14.3) chromosomal translocation, the *Disrupted-in-schizophrenia 1* (*DISC1*) gene has been studied as a key lead to investigate the molecular pathways underlying the pathophysiology of major mental disorders.^1–5^ In addition, perturbations of *DISC1* functions cause behavioral changes in animal models, which are relevant to psychiatric conditions in patients.^1–5^ On the other hand, while genetic studies have identified a large number of risk factor loci,^6–9^ they have not validated *DISC1* as a common risk gene for sporadic cases of schizophrenia defined by the Diagnostic and Statistical Manual of Mental Disorders.^10–12^ Given the intriguing complexity that many of the genetic risk loci found with schizophrenia are shared with other psychiatric diseases,^13–15^ systematic studies with genetically tractable models that address the underlying functional interactions between *DISC1* and psychiatric risk factor genes are warranted.

The fruit fly (*Drosophila melanogaster*) has been used as a powerful model for understanding cellular and molecular mechanisms of neurological disorders.^16,17^ While animal models for mental disorders have empirical and theoretical complications in phenocopying human symptoms, a practical framework for basic research on mental disorders has been proposed as Research Domain Criteria that highlights the importance of elucidating the underlying mechanisms of brain dysfunction at the neurocircuit level.^18–20^ In this framework, mental disorders will be studied at multiple biological and genetic levels using diverse vertebrate and invertebrate models including fruit flies. Accordingly, several works have been reported using the fly model to investigate the mechanisms of mental disorders at the cellular, molecular and genetic levels.^21–28^

**Fig. 1:**
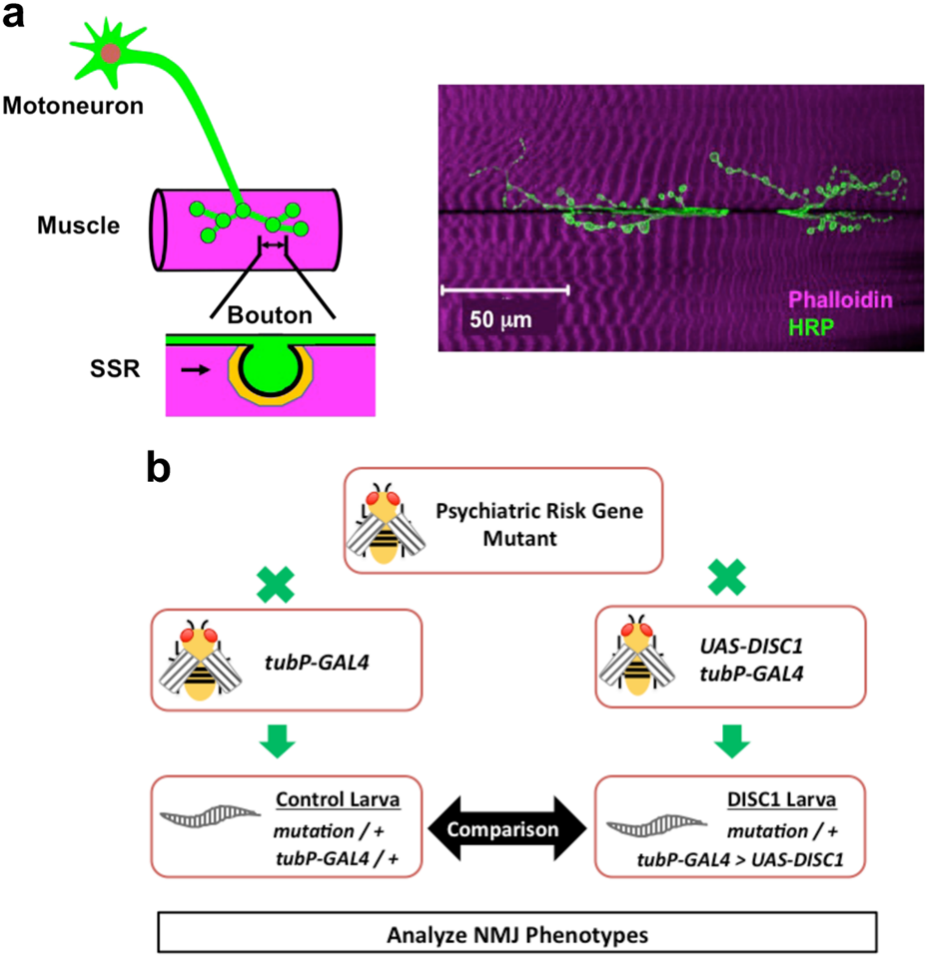
Fruit fly NMJs and screening of interacting genes. **a** Schematic presentation and a confocal image of the fruit fly larval NMJs. The larval NMJs exhibit stereotypic synaptic connections between the identifiable presynaptic motoneuron and the specific postsynaptic muscles. Each of the presynaptic boutons made on the target muscle is surrounded by an intricately convoluted post-synaptic membrane structure called subsynaptic reticulum (SSR), which contains scaffolding proteins and postsynaptic signaling complexes. **b** Screening of interacting genes. Mutant flies (+*/CyO-GFP*; *mutation*/TM6B-GFP) of the fruit fly homologue for a psychiatric risk factor gene are crossed with the control (+/+; *tubP*-*GAL4*/*TM6B-GFP*) or the *DISC1* ^OE^ (*UAS-DISC1*; *tubP-GAL4*/*TM6B-GFP*) flies. The phenotypes of the larval NMJs between the control (+*/*+*; mutation/tubP-GAL4*) and *DISC1* ^OE^ (+*/UAS-DISC1; mutation/tubP-GAL4*) animals were compared

For studying the molecular and genetic mechanisms of synaptogenesis, the *Drosophila* neuromuscular junction (NMJ) is an ideal system. The larval NMJs exhibit stereotypic synaptic connections between the identifiable presynaptic motoneurons and the specific postsynaptic muscles (Fig. 1a).^29–31^ Moreover, the larval NMJs exhibit several important features in common with the excitatory synapses in the vertebrate brain utilizing glutamate as the major neurotransmitter in conjunction with the postsynaptic ionotropic receptors that are homologous to the human glutamate receptors.^29,31,32^ As with the vertebrate central synapse, the synapses on the larval NMJs exhibit a dynamic feature with organized series of boutons that are formed auxiliary or eliminated on the target muscles during development and plasticity.^29,32,33^

To analyze genetic interactions of *DISC1* and psychiatric risk factor genes, we have introduced the human *DISC1* gene in fruit flies to be expressed in their nervous system. We showed previously^27^ that overexpression of *DISC1* (*DISC1* ^OE^) suppresses synaptogenesis at the developing larval NMJs. In this work, we conducted a systematic screening for interacting risk factor genes that cooperatively function with *DISC1* to cause modification of the synaptic phenotypes. We found that *DISC1* interacts with *Neurexin* (*NRXN1*), which encodes a family of synaptic adhesion molecules implicated as a risk factor of various psychiatric disorders including schizophrenia and autism spectrum disorders.

## Results

### Genetic screening of DISC1 interactors in fruit fly NMJs

**Fig. 2:**
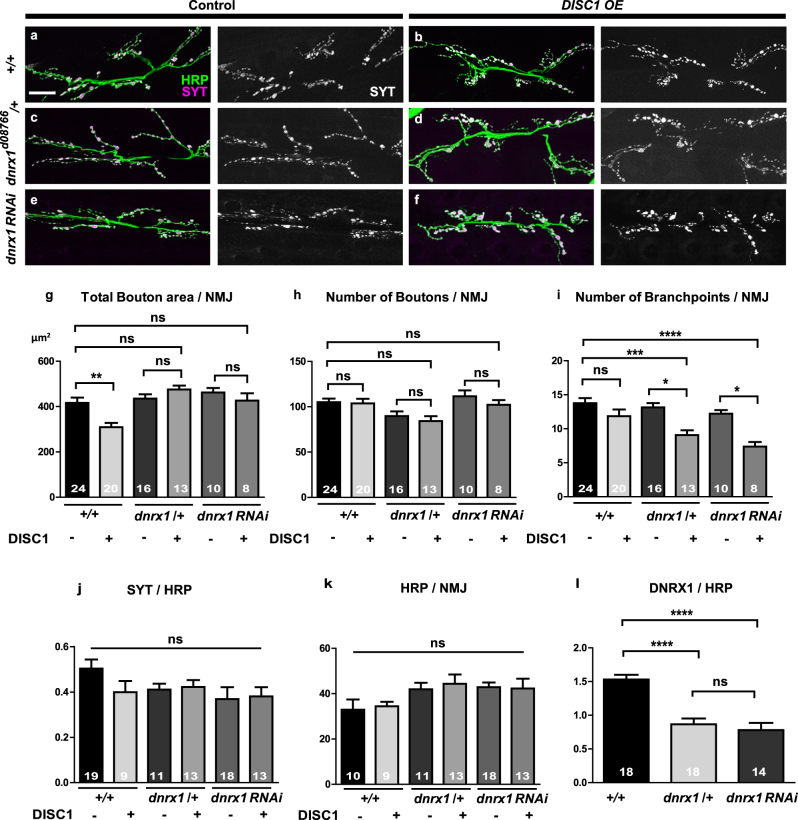
Modification of synaptic morphology with *DISC1* in wild-type and *dnrx1* heterozygous backgrounds. **a**–**f** Representative confocal images. **a**, **b** *w (CS10)* control animals. **c**, **d** *dnrx1* ^d08766^/+ heterozygotes. **e**, **f** *dnrx1 RNAi* driven by *tubP-GAL4*. NMJs on the muscle 6/7 in the second abdominal segment were immunostained with anti-HRP (green) and anti-SYT (magenta) antibodies. Scale bar, 20 μm. **g**–**i** Morphometric analysis of NMJs with (+) or without (−) DISC1 overexpression. **g** Quantification of the total bouton area at the NMJs on the muscle 6/7. **h** Quantification of the number of boutons at the NMJs on the muscle 6/7. **i** Quantification of the number of axonal branch points at the NMJs on the muscle 6/7. **j** Quantification of SYT expression level normalized to HRP. **k** Quantification of HRP immunoreactivity. **l** Quantification of DNRX1 expression level in *dnrx1* ^d08766^ heterozygous and RNAi NMJs. Data are means ± SEM. *p < 0.05, **p < 0.01, ***p < 0.001, and ****p < 0.0001 by one-way ANOVA followed by the Tukey’s pot hoc test. Number of each sample is indicated at the bottom of the bar. The statistical values are listed in Supplementary Table 1

To analyze the synaptic morphology, we performed immunological staining of larval NMJs using a pan-neuronal antibody, anti-horseradish peroxidase protein (HRP), and a synaptic vesicle antibody, anti-Synaptotagmin (SYT), and determined the total bouton area, the number of boutons, and the number of axonal branch points that are made on the muscle 6/7 in the second abdominal segment of early third instar larvae (116–120 h after egg laying). Consistent with the previous study,^27^ *DISC1* ^OE^ caused a reduction in total bouton area (analysis of variance (ANOVA) F (5, 85) = 7.49, *p* < 0.0001, +/+ DISC1 (−) vs. +/+ DISC1 (+), *p* = 0.0021, by Tukey’s post hoc test) (Fig. 2g) but not the numbers of boutons (ANOVA F (5, 82) = 3.19, *p* = 0.0111, +/+ DISC1 (−) vs. +/+ DISC1 (+), *p* = 0.9216, by Tukey’s post hoc test) (Fig. 2h) and axonal branch points (ANOVA F (5, 84) = 7.08, *p* < 0.0001, +/+ DISC1 (−) vs. +/+ DISC1 (+), *p* = 0.1536, by Tukey’s post hoc test) (Fig. 2i) in the wild-type background. Based on this anatomical phenotype, we then performed a genetic screening for psychiatric risk gene mutations that modified the *DISC1* ^OE^ synaptic phenotype. Briefly, we expressed *DISC1* in the heterozygous background of the fly mutations and compared their synaptic phenotypes against the *DISC1* ^OE^ phenotype in the wild-type background (Fig. 1b).

Among the genes identified in this screening, a mutation of *dnrx1* (*dnrx1* ^d08766^), the *Drosophila* homolog^34–39^ of the human *Neurexin* (*NRXN1*), caused an modification of the *DISC1* ^OE^ phenotype in the developing NMJs (Fig. 2a–i). Although the *dnrx1* ^d08766^ mutation did not alter synaptic structures in the heterozygous background (total bouton area: ANOVA F (5, 85) = 7.49, *p* < 0.0001, +/+ DISC1 (−) vs. dnrx1/+ DISC1 (−), *p* = 0.9853, by Tukey’s post hoc test) (Fig. 2g) (number of boutons: ANOVA F (5, 82) = 3.19, *p* = 0.0111, +/+ DISC1 (−) vs. dnrx1/+ DISC1 (−), *p* = 0.0901, by Tukey’s post hoc test) (Fig. 2h) (number of branch points: ANOVA F (5, 84) = 7.08, *p* < 0.0001,+/+ DISC1 (−) vs. dnrx1/+ DISC1 (−), *p* = 0.9265, by Tukey’s post hoc test) (Fig. 2i), it failed *DISC1* ^OE^ to suppress synaptic bouton area in the heterozygous background (ANOVA F (5, 85) = 7.49, *p* < 0.0001, dnrx1/+ DISC1 (−) vs. dnrx1/+ DISC1 (+), *p* = 0.8366, by Tukey’s post hoc test) (Fig. 2g). Moreover, *DISC1* ^OE^ caused reductions in the number of axonal branch points in the *dnrx1* ^d08766^ */*+ heterozygous background (ANOVA F (5, 84) = 7.08, *p* < 0.0001, dnrx1/+ DISC1 (−) vs. dnrx1/+ DISC1 (+), *p* = 0.0333, by Tukey’s post hoc test) (Fig. 2i) resulting in a significant suppression from the wild type (*p* < 0.0001, +/+ DISC1 (−) vs. dnrx1/+ DISC1 (+), *p* = 0.0009, by Tukey’s post hoc test) (Fig. 2i). On the other hand, although the group as a whole shows a difference (ANOVA F (5, 82) = 3.19, *p* = 0.0111), *DISC1* ^OE^ did not alter the numbers of the synaptic boutons in both the wild-type (+/+ DISC1 (−) vs. +/+ DISC1 (+), *p* = 0.9216, by Tukey’s post hoc test) and the *dnrx1* ^d08766^ */*+ heterozygous backgrounds (dnrx1/+ DISC1 (−) vs. dnrx1/+ DISC1 (+), *p* = 0.9993, by Tukey’s post hoc test) (Fig. 2h).

To further investigate the genetic interaction between *DISC1* and *dnrx1*, we analyzed whether a similar modification of the *DISC1* ^OE^ synaptic phenotype was caused by a partial suppression of *DNRX1* by RNA interference (RNAi). One of the RNAi lines we tested, *P{TRiP. JF02652}*, exhibited approximately 50% downregulation of the DNRX1 protein level (ANOVA F (2, 47) = 22.89, *p* < 0.0001, +/+ vs. dnrx1 RNAi, *p* < 0.0001, by Tukey’s post hoc test) (Fig. 2l), which was comparable to the downregulation observed in the *dnrx1* ^d08766^ */*+ heterozygotes (dnrx1/+ vs. dnrx1 RNAi, *p* = 0.7833, by Tukey’s post hoc test) (Fig. 2l). As was the case for the *dnrx1* ^d08766^ */*+ heterozygotes, *dnrx1* RNAi did not alter the synaptic morphology on its own (total bouton area: ANOVA F (5, 85) = 7.49, *p* < 0.0001, +/+ DISC1 (−) vs. dnrx1 RNAi DISC1 (−), *p* = 0.7443, by Tukey’s post hoc test) (Fig. 2g) (number of boutons: ANOVA F (5, 82) = 3.19, *p* = 0.0111, +/+ DISC1 (−) vs. dnrx1 RNAi DISC1 (−), *p* = 0.9909, by Tukey’s post hoc test) (Fig. 2h) (number of branch points: ANOVA F (5, 84) = 7.08, *p* < 0.0001, +/+ DISC1 (−) vs. dnrx1 RNAi DISC1 (−), *p* = 0.7223, by Tukey’s post hoc test) (Fig. 2i). Moreover, *DISC1* ^OE^ with *dnrx1* RNAi failed to reduce the total bouton area (ANOVA F (5, 85) = 7.49, *p* < 0.0001, dnrx1 RNAi DISC1 (−) vs. dnrx1 RNAi DISC1 (+), *p* = 0.9569, by Tukey’s post hoc test) (Fig. 2g) but caused a significant reduction in the number of axonal branch points (ANOVA F (5, 84) = 7.08, *p* < 0.0001, dnrx1 RNAi DISC1 (−) vs. dnrx1 RNAi DISC1 (+), *p* = 0.0276, by Tukey’s post hoc test) (Fig. 2i), recapitulating the modification of the *DISC1* ^OE^ synaptic phenotype in the *dnrx1* ^d08766^ */*+ heterozygotes.

To examine whether *DISC1* ^OE^ altered the expression of the immunological markers used in the anatomical analyses, we quantitated the signal intensities of SYT and HRP. The expression level of neither protein was altered with *DISC1* ^OE^ in the wild-type, *dnrx1* ^d08766^ */*+, nor RNAi backgrounds (SYT: ANOVA F (5, 68) = 0.22, *p* = 0.9550) (Fig. 2j) (HRP: ANOVA F (5, 68) = 1.72, *p* = 0.1423) (Fig. 2k).

### DISC1 stimulates active zone density in wild-type but not in *dnrx1/+* background

Neurexins are a family of synaptic adhesion molecules expressed on presynaptic neurons and organize the formation and maturation of both presynaptic and postsynaptic structures through interactions with postsynaptic partners, such as Neuroligins (NLGs).^40–42^ In the fly NMJs, DNRX1 mostly localizes to the active zone of presynaptic terminals and controls the formation of active zone and postsynaptic structures.^34–39^

**Fig. 3:**
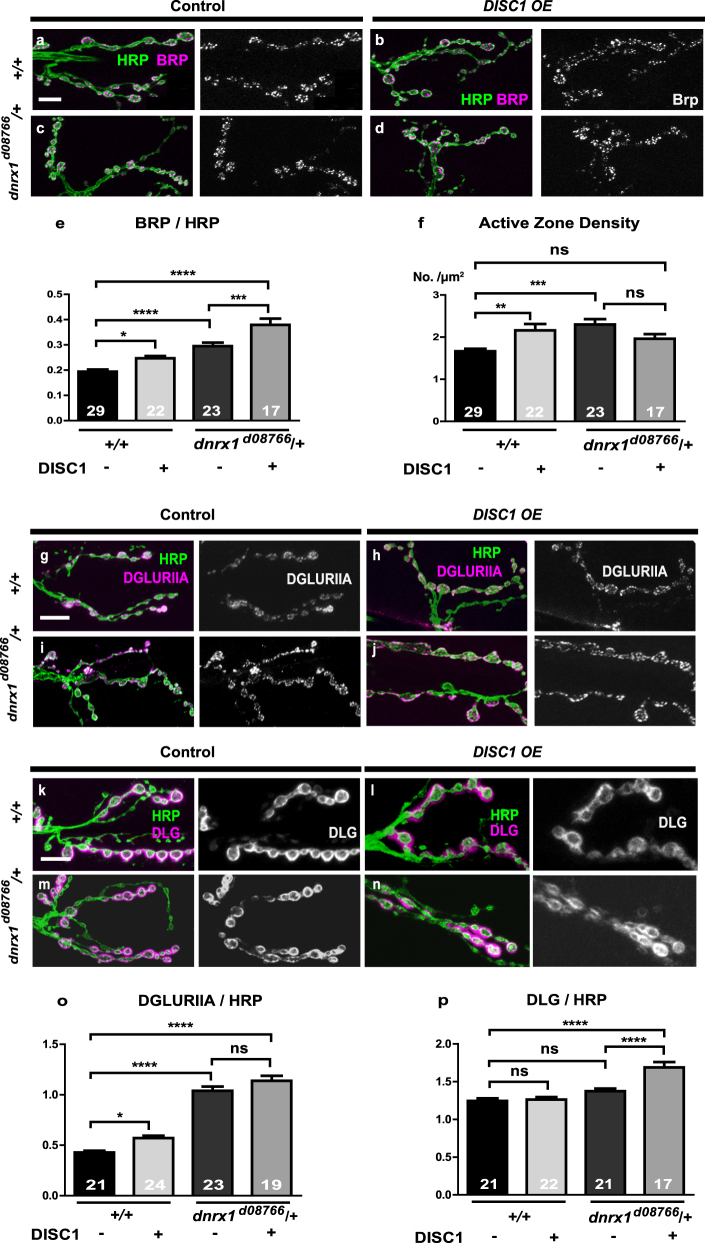
Expression of presynaptic and postsynaptic proteins in NMJ boutons. **a**–**d** Active zone formation with or without DISC1 overexpression. Representative confocal images. **a**, **b** *w (CS10)* control animals. **c**, **d** *dnrx1* ^d08766^/+ heterozygotes. Larval NMJs were immunostained with anti-HRP (green) and anti-BRP (magenta) antibodies. Scale bar, 20 μm. **e** Quantification of BRP expression level in the muscle 6/7 boutons normalized to HRP immunoreactivity. **f** Quantification of active zone density as determined by the number of BRP puncta per bouton area. **g**–**j** Expression of DGLURIIA with or without DISC1 overexpression. Representative confocal images. **g**, **h** *w (CS10)* control animals. **i**, **j** *dnrx1* ^d08766^/+ heterozygotes. Larval NMJs were immunostained with anti-HRP (green) and anti-DGLURIIA (magenta) antibodies. Scale bar, 20 μm. **k**–**n** Expression of DLG with or without DISC1 overexpression. Representative confocal images. **k**, **l** *w (CS10)* control animals. **m**, **n** *dnrx1* ^d08766^/+ heterozygotes. Larval NMJs were immunostained with anti-HRP (green) and anti-DLG (magenta) antibodies. Scale bar, 20 μm. **o** Quantification of DGLURIIA expression level in the muscle 6/7 boutons normalized to HRP immunoreactivity. **p** Quantification of DLG expression level in the muscle 6/7 boutons normalized to HRP immunoreactivity. Data are means ± SEM. *p < 0.05, **p < 0.01, ***p < 0.001, and ****p < 0.0001 by one-way ANOVA followed by the Tukey’s pot hoc test. Number of each sample is indicated at the bottom of the bar. The statistical values are listed in Supplementary Table 1

To further analyze the functional interactions of *dnrx1* and *DISC1* in synaptogenesis, we examined active zone formation using a presynaptic marker, Bruchpilot (BRP), which is the fly homolog of the vertebrate ELKS/CAST active zone proteins essential for rapid synaptic vesicle release.^43–46^ In the wild-type, *DISC1* ^OE^ stimulated the BRP level (ANOVA F (3, 87) = 32.73, *p* < 0.0001, +/+ DISC1 (−) vs. +/+ DISC1 (+), *p* = 0.02, by Tukey’s post hoc test) (Fig. 3e) and the active zone density (ANOVA F (3, 96) = 7.22, *p* *=* 0.0002, +/+ DISC1 (−) vs. +/+ DISC1 (+), *p* = 0.0049, by Tukey’s post hoc test) (Fig. 3f). Both the BRP level (ANOVA F (3, 87) = 32.73, *p* < 0.0001, +/+ DISC1 (−) vs. dnrx1/+ DISC1 (−), *p* < 0.0001, by Tukey’s post hoc test) (Fig. 3e) and the active zone density (ANOVA F (3, 96) = 7.22, *p* *=* 0.0002, +/+ DISC1 (−) vs. dnrx1/+ DISC1 (−), *p* *=* 0.0003, by Tukey’s post hoc test) (Fig. 3f) were increased in the *dnrx1* ^d08766^ */*+ heterozygous (DISC1 minus) yet *DISC1* ^OE^ further stimulated the BRP level resulting in a significant increase from the wild-type (+/+ DISC1 (−) vs. dnrx1/+ DISC1 (+), *p* < 0.0001, by Tukey’s post hoc test) (Fig. 3e). By contrast, *DISC1* ^OE^ failed to increase the active zone density in the *dnrx1* ^d08766^ */*+ heterozygous background (dnrx1/+ DISC1 (−) vs. dnrx1/+ DISC1 (+), *p* *=* 0.2355, by Tukey’s post hoc test) (Fig. 3f).

### DISC1 stimulates glutamate receptor expression in wild-type but not in *dnrx1/+* background

In addition to presynaptic structures, Neurexins control postsynaptic structures via trans-synaptic interaction with its partner molecules.^40–42^ In particular, presynaptic Neurexins trans-synaptically control postsynaptic *α*-amino-3-hydroxyl-5-methyl-4-isoxazole-propionate (AMPA) glutamate receptor stabilization through the interactions with postsynaptic binding partners, such as leucine-rich-repeat-transmembrane-neuronal 2 protein and NLG.^47^

To determine whether reduction of *dnrx1* activity modified the *DISC1* ^OE^ phenotype in post-synaptic cells, we investigated the expression of Drosophila-glutamate-receptor-IIA (DGLURIIA), one of the subunits of the *Drosophila* AMPA receptor postsynaptically expressed at the larval NMJs.^48–50^ Of note, *DISC1* ^OE^ stimulated the DGLURIIA level in the wild-type (ANOVA F (3, 83) = 96.4, *p* < 0.0001, +/+ DISC1 (−) vs. +/+ DISC1 (+), *p* = 0.0216, by Tukey’s post hoc test) (Fig. 3o) but not in the *dnrx1* ^d08766^ */*+ heterozygous background (dnrx1/+ DISC1 (−) vs. dnrx1/+ DISC1 (+), *p* = 0.2194, by Tukey’s post hoc test) (Fig. 3o), which resulted in a significant increase in the DGLURIIA level on its own (Fig. 3g–j) (+/+ DISC1 (−) vs. dnrx1/+ DISC1 (−), *p* < 0.0001, by Tukey’s post hoc test) (Fig. 3o).

### DISC1 causes mislocalization of a postsynaptic density marker in *dnrx1/+* background

**Fig. 4:**
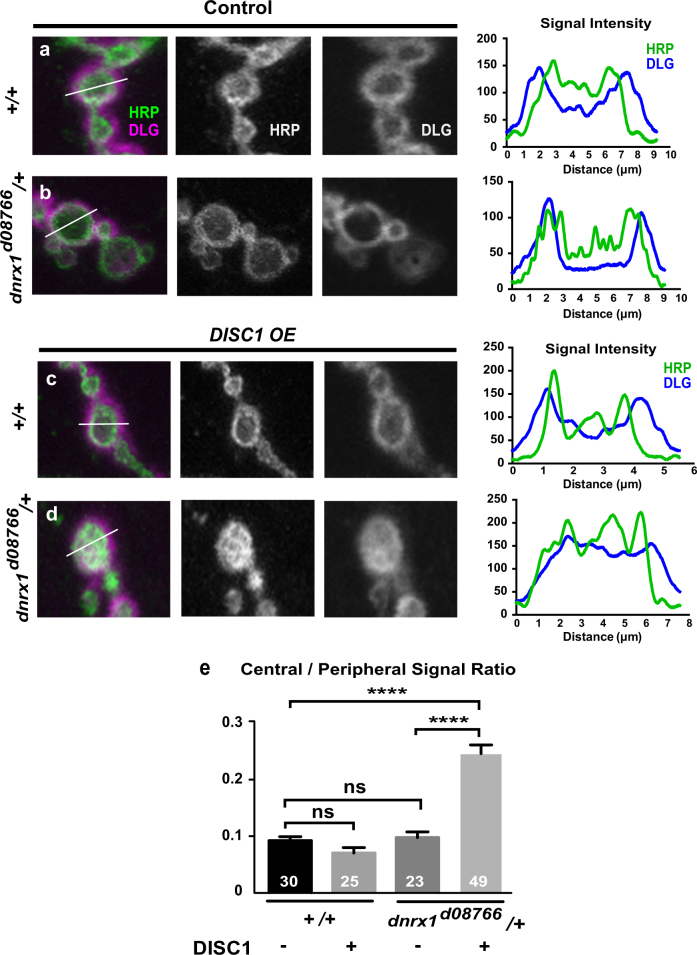
Quantitative analysis of DLG localization in NMJ boutons. **a**–**d** Representative *DISC1* ^OE^ bouton images in the control and *dnrx1* ^d08766^/+ heterozygous larvae. Larval NMJ boutons were immunostained with anti-HRP (green) and anti-DLG (magenta) antibodies. Right panels show quantification of fluorescence signal intensity along the lines indicated in **a**–**d**. **e** Quantification of the central/peripheral ratio of the DLG signals in the NMJ boutons. Data are means ± SEM. ****p < 0.0001 by one-way ANOVA followed by the Tukey’s pot hoc test. Number of each sample is indicated at the bottom of the bar. The statistical values are listed in Supplementary Table 1

To further investigate the *DISC1*-*dnrx1* interaction, we examined the postsynaptic density specialization by immunological staining for Discs large (DLG), a fruit fly homolog of the mammalian MAGUK proteins, SAP 97, SAP102, and PSD-95, that are critical for postsynaptic assembly at glutamatergic synapses.^51,52^ It has been shown that nul mutations of *dnrx1* alter subcellular distribution of DLG in the postsynaptic cells of the fly NMJs.^35^ In the fly NMJs, DLG localizes to an intricately convoluted post-synaptic membrane structure called subsynaptic reticulum (Figs. 1a and 3k), which contains scaffolding proteins and postsynaptic signaling complexes. While *DISC1* ^OE^ failed to stimulated DLG expression in wild-type background (ANOVA F (3, 77) = 20.8, *p* < 0.0001, +/+ DISC1 (−) vs. +/+ DISC1 (+), *p* = 0.9911, by Tukey’s post hoc test) (Fig. 3p), it upregulated the DLG level in the *dnrx1* ^d08766^ */*+ heterozygous background (dnrx1/+ DISC1 (−) vs. dnrx1/+ DISC1 (+), *p* < 0.0001, by Tukey’s post hoc test) (Fig. 3p). Moreover, *DISC1* ^OE^ caused diffuse DLG localization in the *dnrx1* ^d08766^ */*+ heterozygous background (ANOVA F (3, 122) = 45.4, *p* < 0.0001, +/+ DISC1 (−) vs. dnrx1/+ DISC1 (+), *p* < 0.0001, by Tukey’s post hoc test) (Fig. 4) while normal peripheral DLG localization was maintained in both *dnrx1* ^d08766^ */*+ (DISC1 minus) (+/+ DISC1 (−) vs. dnrx1/+ DISC1 (−), *p* = 0.99, by Tukey’s post hoc test) and *DISC1* ^OE^ in the wild-type background (+/+ DISC1 (−) vs. dnrx1/+ DISC1 (−), *p* = 0.9939, +/+ DISC1 (−) vs. +/+ DISC1 (+), *p* < 0.7128, by Tukey’s post hoc test) (Fig. 4e).

### DISC1 causes locomotor defects in *dnrx1/+* background

To analyze the behavioral consequence of the alterations observed at the NMJs, we examined larval locomotor activity (Supplementary Table S1). Although *DISC1* ^OE^ did not cause significant effect on the average locomotion speed in the wild-type background (ANOVA F (3, 75) = 5.798, *p* = 0.0013, +/+ DISC1 (−) vs. +/+ DISC1 (+), *p* = 0.194, by Tukey’s post hoc test) (Supplementary Fig. S1A), it caused significant reduction in the average locomotion speed in the *dnrx1* ^d08766^ */*+ heterozygous background (+/+ DISC1 (−) vs. dnrx1/+ DISC1 (+), *p* = 0.0037, by Tukey’s post hoc test) (Supplementary Fig. S1A). Similarly, *DISC1* ^OE^ did not alter peak locomotion speed (highest speed marked in 1 min measurement) in the wild-type background (ANOVA F (3, 75) = 8.879, *p* < 0.0001, +/+ DISC1 (−) vs. +/+ DISC1 (+), *p* = 0.1031, by Tukey’s post hoc test) (Supplementary Fig. S1B) but caused significant reduction in the average locomotion speed in the *dnrx1* ^d08766^ */*+ heterozygous background (+/+ DISC1 (−) vs. dnrx1/+ DISC1 (+), *p* = 0.0009, by Tukey’s post hoc test) (Supplementary Fig. S1B).

Despite the diverse alterations at the NMJs and in the larval locomotor activity, no difference was detected in the cell body size in the ventral nerve cord (Supplementary Fig. S2A–D) (ANOVA F (3, 189) = 2.04, *p* = 0.1101) (Supplementary Fig. S2E), suggesting that the observed changes are not the consequences of the undergrowth of the cognate motoneurons.

### Presynaptic overexpression of DISC1 suppresses DNRX1 in NMJ boutons

**Fig. 5:**
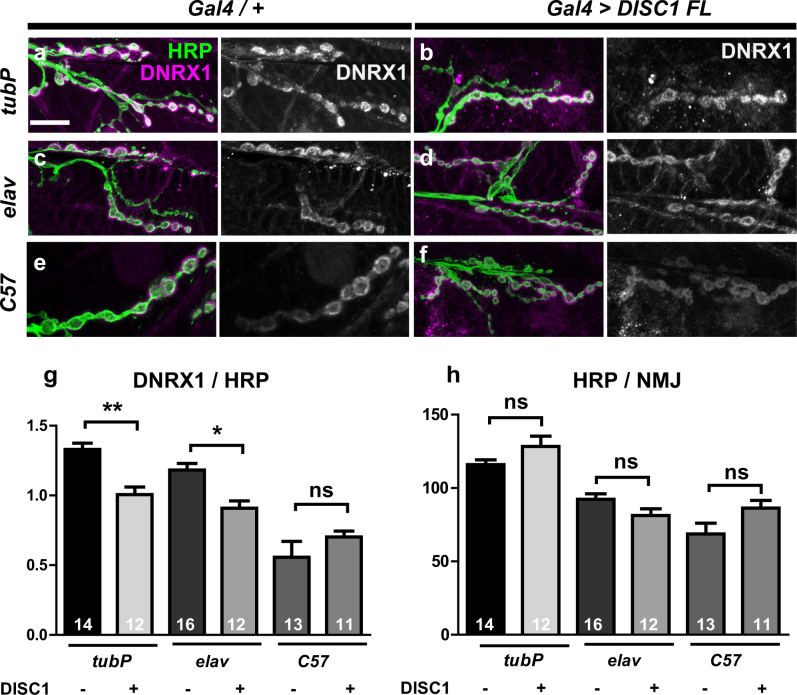
Suppression of DNRX1 with DISC1. **a**–**f** Representative confocal images of wild-type NMJs with or without DISC1 overexpression. **a**, **b** Ubiquitous expression with *tubP-GAL4*. **c**, **d** Pre-synaptic expression with *elav-GAL4*. **e**, **f** Post-synaptic expression with *C57-GAL4*. **a**, **c**, **e** Control NMJs without *DISC1* expression. **b**, **d**, **f** NMJs with DISC1 overexpression driven by the designated GAL4 driver. Synaptic boutons at the NMJs on the muscle 6/7 in the second abdominal segment were immunostained with anti-HRP (green) and anti-DNRX1 (magenta) antibodies. Scale bar, 20 μm. **g** Quantification of DNRX1 expression level in the muscle 6/7 boutons normalized to HRP immunoreactivity. **h** Quantification of HRP immunoreactivity in the muscle 6/7 boutons. Data are means ± SEM. Data are means ± SEM. *p < 0.05, **p < 0.01 by one-way ANOVA followed by the Tukey’s pot hoc test. Number of each sample is indicated at the bottom of the bar. The statistical values are listed in Supplementary Table 1

The result that *DISC1* ^OE^ caused mislocalization of a postsynaptic density marker in the *dnrx1/*+ background in part mimicked the *dnrx1* phenotype and prompted us to address whether *DISC1* suppressed the DNRX1 protein level in the synaptic boutons. Intriguingly, *DISC1* ^OE^ with a ubiquitous driver (*tubP-GAL4*) caused moderate but significant reduction in the DNRX1 level (*tubP* DISC1 (−) vs. *tubP* DISC1 (+), *p* = 0.009, by Tukey’s post hoc test) (Fig. 5g) while the expression level of the pan-neuronal marker HRP remained unchanged (*tubP* DISC1 (−) vs. *tubP* DISC1 (+), *p* = 0.5606, by Tukey’s post hoc test) (Fig. 5h). To determine whether presynaptic or postsynaptic *DISC1* ^OE^ caused downregulation of DNRX1, we then expressed *DISC1* using either a neuron-specific (*elav-GAL4*), or a muscle-specific (*C57-GAL4*) driver (Fig. 5g) and found that neuron-specific but not muscle-specific *DISC1* ^OE^ downregulated the DNRX1 level (*elav* DISC1 (−) vs. *elav* DISC1 (+), *p* = 0.0331; *C57* DISC1 (−) vs. *C57* DISC1 (+), *p* = 0.6596, by Tukey’s post hoc test).

### Axonal localization of the DISC1 protein is crucial for efficient suppression of DNRX1

**Fig. 6:**
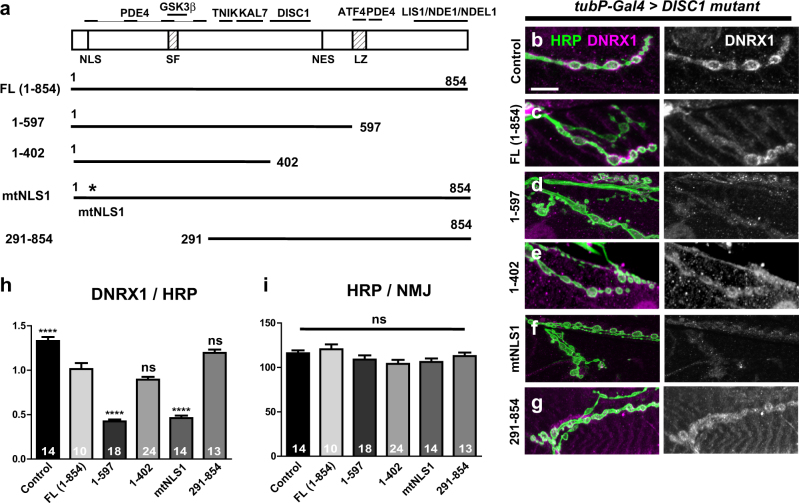
Suppression of DNRX1 with deletion/mutation DISC1 constructs. **a** DISC1 protein domains and the structure of the deletion/mutation constructs. *NLS* nuclear localization signal, *SF* Ser-Phe rich domain, *NES* nuclear exclusion signal, *LZ* leucine-zipper domain. Representative interacting proteins are shown above the structure. *PDE4* phosphodiesterase type 4, *GSK3β* glycogen synthase kinase 3β, *TNIK* TRAF2 and NCK-interacting protein kinase, *KAL7* kalirin 7, *ATF4* activating transcription factor 4, *LIS1* lissencephaly protein 1, *NDE1* nuclear distribution protein nudE homolog 1, *NDEL1* nuclear distribution protein nudE-like 1. **b**–**g**) Representative confocal images of NMJs immunostained with anti-HRP (green) and anti-DNRX1 (magenta) antibodies. The deletion/mutation DISC1 proteins were driven by *tubP-GAL4*. **h** Quantification of DNRX1 expression level in the muscle 6/7 boutons normalized to HRP immunoreactivity. Comparisons are against FL (1–854). Note that both 1–402 and 291–854 caused DNRX1 suppression as did FL (1–854) (control vs. FL (1–402), *p* = 0.0001; control vs. 291–854, *p* = 0.0108, by Dunnett’s post hoc test). **i** Quantification of HRP immunoreactivity in the muscle 6/7 boutons. Data are means ± SEM. *p < 0.05, **p < 0.01, ***p < 0.001, with one-way ANOVA followed by the Dunnett’s post hoc test. Number of each sample is indicated at the bottom of the bar. The statistical values are listed in Supplementary Table 1

To analyze the underlying mechanism of the suppression of DNRX1, we expressed a series of DISC1 deletion constructs^27^ (Fig. 6a) and assessed the DNRX1 protein level in synaptic boutons (Fig. 6b–h). Intriguingly, DISC1 (1–597), which corresponds to the Scottish family truncation with a prominent axonal localization,^27^ exhibited stronger suppression of DNRX1 than the full-length DISC1 (ANOVA F (5, 87) = 100.6, *p* < 0.0001, FL (1–854) vs. 1–597, *p* = 0.0001, by Dunnett’s post hoc test against FL) (Fig. 6d, h), while further removal of the protein domains (DISC1(1–402)) reverted the suppressing activity similar to the full-length (FL(1–854)) protein level (FL (1–854) vs. 1–402, *p* = 0.1108, by Dunnett’s post hoc test against FL) (Fig. 6e, h). Notably, DISC1 (1–402) lacks the nuclear export signal with weak axonal localization,^27^ suggesting the importance of axonal targeting over nuclear localization for the suppression of the synaptic DNRX level. Consistently, DISC1 (mtNLS1), which is exclusively localized to the cytoplasm with robust axonal targeting,^22,27^ exhibited strong DNRX1 suppression (FL (1–854) vs. mtNLS1, *p* = 0.0001, by Dunnett’s post hoc test against FL) (Fig. 6f, h) while further removal of the amino-terminal domains (DISC1 (291–854)) including the PDE4 and GSK3β binding motifs reverted the suppressing activity similar to the full-length protein level (FL (1–854) vs. 291–854, *p* = 0.0688, by Dunnett’s post hoc test against FL) (Fig. 6g, h). On the other hand, none of the DISC1 derivatives caused an alteration in the expression level of the pan-neuronal marker HRP used as an internal control (ANOVA F (5, 87) = 1.79, *p* = 0.1224) (Fig. 6i).

## Discussion

In this paper, we have shown that *DISC1* interacts with a psychiatric risk factor gene, *dnrx1*, the *Drosophila* homolog of the human *NRXN1*,^40–42^ in the glutamatergic synapses on the larval NMJs. While *DISC1* ^OE^ upregulated the expression of the ELKS/CAST protein BRP^43–46^ in presynaptic neurons in both the wild-type and the *dnrx1* heterozygous backgrounds, reduction of *dnrx1* suppressed *DISC1*-mediated stimulation of active zone density. *DISC1* ^OE^ also upregulated expression of DGLURIIA, a component of the AMPA receptor expressed postsynaptically in the fly muscle,^48–50^ but failed to do so in the *dnrx1* heterozygous background. On the other hand, reduction of *dnrx1* potentiated *DISC1* to stimulate the expression of DLG, the *Drosophila* homolog of PSD-95, which controls postsynaptic density assembly.^51,52^ Moreover, *DISC1* ^OE^ caused diffuse DLG localization in the *dnrx1* ^d08766^ */*+ heterozygous background. We have also shown that *DISC1* ^OE^ in presynaptic but not postsynaptic cells suppressed the DNRX1 expression in the synaptic boutons. Analyses with a series of DISC1 domain deletions have revealed that removal of a carboxyl-terminal domain (DISC1 (1–597),^27^ which corresponds to the Scottish family truncation, resulted in stronger suppression of DNRX1 than the full-length protein. Likewise, a mutation of the nuclear localization signal (mtNLS1), which leads to exclusive cytoplasmic localization of the DISC1 protein with robust axonal targeting,^27^ resulted in a stronger suppression.

Increasing lines of evidences suggest that aberrant synaptic development and plasticity have important roles in the etiology of various mental disorders.^7–9,53^ In this study, we have found that *dnrx1* exhibits functional interactions with *DISC1* in the glutamatergic synapses at the larval NMJs. Notably, the observed mislocalization of DLG caused by *DISC1* ^OE^ in the *dnrx1* ^d08766^ */*+ background is reminiscent of the mislocalization phenotype described for *dnrx1* and *dnlg1* double mutants.^35^ In addition, we have also identified *dnlg1*,^54,55^ the fruit fly homolog of the human NLG1,^40–42,56,57^ as another interacting risk factor gene that modifies the functions of *DISC1* in glutamatergic synapses (P. H. and K. F. T., in preparation).

Although we have shown that partial reductions of the *dnrx1* activity led to modification of the *DISC1* ^OE^ synaptic phenotypes both at the morphological and molecular levels, we have not been able to show direct interaction between the DNRX1 and DISC1 proteins. Since the comprehensive DISC1 interactome studies also fail to identify NRXN1 as a direct interacting partner,^3,4,58–60^ we would rather speculate complex converging interactions of *DISC1* and *NRXN1* in glutamatergic synapses involving trans-synaptic interactions between the presynaptic and postsynaptic cells that cause a partial suppression of the DNRX1 protein level in the boutons. In line with this notion, a recent study^61^ suggests that Neurexin–NLG complex might regulate the DISC1-containing Kalirin-7/Rac1 (RAS-related C3-botulinum toxin substrate 1) signal complex through the interaction of Kalirin-7 and NLG. Further studies are warranted to examine the interaction between the NRXN1 and DSIC1 proteins in the nervous system development.

Mediating adhesive interactions between presynaptic and postsynaptic cells, Neurexins and NLGs are critical molecules for the precise organization and alignment of synaptic compartments and molecular complexes.^40–42^ In presynaptic cells, Neurexins bind directly to the scaffolding proteins CASK (calcium/calmodulin dependent serine protein kinase) and MINT1 (Munc-18-interacting 1) via PDZ (PSD-95 DLG Zonula occludens 1) domain interactions, and indirectly recruit elements of the presynaptic release machinery.^40,41^ Presynaptic Neurexins trans-synaptically control postsynaptic AMPA receptor stabilization through interaction with its postsynaptic partners such as LRRTM2 and NLGs,^47^ which in turn interact with PDZ domain proteins such as PSD-95 in postsynaptic neurons.^40,41^ It has been shown that DISC1 regulates postsynaptic spine morphology and AMPA-type glutamate receptor expression via interaction with PSD-95.^59,60^ It is also noteworthy that the expression of NRXN1 and NRXN3 are dysregulated in a mutant mouse line carrying an L100P DISC1 missense mutation.^62^ These results as a whole suggest an intriguing convergence of intracellular signaling networks mediated by DISC1 and NRXN1 in the development and plasticity of glutamatergic synapses.

*NRXN1* has been identified as a risk factor gene for diverse psychiatric disorders including schizophrenia and autism spectrum disorders.^40,63,64^ By analyzing the genetic interactions in fruit fly glutamatergic synapses, we have identified a novel interaction between DISC1 and a synaptic cell adhesion molecule that organizes trans-synaptic structures and functions. On the other hand, it should be noted that our study utilized a gain-of-function approach expressing the human DISC1 protein in a heterologous background. Further studies including loss-of-function studies in mammalian models are warranted as are epistasis studies of human subjects. Recent progress using patient-derived induced pluripotent cells^65,66^ would also help to identify the molecular process co-regulated by *NRXN1* and *DISC1* involved in the pathophysiology of neuropsychiatric abnormalities.

## Materials and methods

### Fly stocks

A *white* (*w*) stock ten times outcrossed with *Canton S* (*w (CS10)*) was used as the standard stock. Construction of transgenic flies carrying *UAS-DISC1* transgene including *DISC1 (1-597)* and *DISC1 (mNLS1)* has been described previously.^22,27^ To ensure homogeneous genetic background, all fly stocks were outcrossed to *w (CS10)* at least five times. The following stocks were obtained from the Bloomington Stock Center (Bloomington, IN, USA): *dnrx1* ^d08766^, *dnrx1* RNAi *P{TRiP. JF02652}*, and *GAL4* drivers (*tubP-GAL4*, *elav-GAL4*, and *C57*-*GAL4*). All stocks were raised at 25 °C on a standard fly food.

### Genetic screening

For the screening, mutant lines were balanced with a double balancer stock (*w/w; Sp / CyO Act-GFP; Pr Dr/ TM6B ubi-GFP*). The resulting progeny carrying the mutation were then crossed either with control (*w;*+*; tubP-GAL4/TM6B ubi-GFP*) or with *DISC1* ^*OE*^ (*w; UAS-DISC1(CS10)6-6(II); tubP-GAL4/ TM6B ubi-GFP*) flies. Larvae were raised at 25 ˚C, and non-GFP animals, which carry the *tubP-GAL4* chromosome, were selected for dissection. Details of the genetic scheme are available upon request.

### Immunohistochemistry

Mouse anti-SYT monoclonal antibody (3H2 2D7) was obtained from the Developmental Studies Hybridoma Bank (DSHB) (University of Iowa, IA, USA) and used at 1:2 dilution. The anti-SYT (3H2 2D7) was originally developed and deposited to the DSHB by Kai Zinn (Caltech), and its specificity is described in Dubuque, et al.^67^ and Yoshihara and Littleton.^68^ Mouse anti-DGLURIIA monoclonal antibody (8B4D2) was obtained from DSHB and used at 1:50 dilution. The anti-DGLURIIA (8B4D2) was originally developed and deposited to the DSHB by Corey Goodman (Stanford University), and its specificity is described in Marrus, et al.^69^ Mouse anti-BRP monoclonal antibody (NC82) was obtained from DSHB and used at 1:20 dilution. The anti-BRP (NC82) was originally developed and deposited to the DSHB by Eric Buchner (Theodor-Boveri-Institute für Biowissenschaften, Germany), and its specificity is described in Wagh, et al.^44^ and Kittel, et al.^43^ Mouse anti-DLG monoclonal antibody (4F3) was obtained from DSHB and used at 1:3 dilution. The anti-DLG (4F3) was originally developed and deposited to the DSHB by Corey Goodman (Stanford University), and its specificity has been described in Parnas, et al.^70^. The rabbit anti-DNRX1 antibody was originally developed and provided by David Featherstone (university of Illinois) and used at 1:100 dilution. The specificity of the anti-DNRX1 is described in Chen, et al.^36^ including the immunoreactivity tests against the NMJs in *dnrx1* null mutants. Pan-neural anti-HRP conjugated with fluorescein-isothiocyanate (Jackson ImmunoResearch, West Grove, PA, USA) was used at 1:100 dilution, and Alexa-conjugated secondary antibodies (Molecular probes, Eugene, OR, USA) were used at 1:1000 dilution. Confocal images were captured with Zeiss LSM510 or LSM710 microscope.

### Quantification of NMJ structure and fluorescence intensity

For quantification of synaptic phenotypes, we raised larvae at 25 ˚C and fixed at 116–120 h after egg laying and then analyzed the larval longitudinal muscles 6/7 in the abdominal hemisegment A2 according to the method described previously.^71^ Anti-HRP and anti-SYT were used to label the neuronal termini and synaptic boutons, respectively. Total bouton area was determined using Image-J (http://rsb.info.nih.gov/ij/) based on anti-SYT immunoreactivity. Protein expression levels were determined with Image-J based on fluorescent intensities in the boutons using the control and test samples processed simultaneously in the same tube. Confocal images were captured using identical settings. Anti-HRP immunoreactivity was used as an internal control.

### Larval locomotion analysis

Wandering third instar larvae were harvested from vials using a paint brush. The larvae were rinsed with DW and transferred to an agar plate using a paint brush. One larva at a time was transferred to a freshly prepared 90 mm agar plate and acclimatized until it started forward peristalsis, then larval locomotion was filmed for 1 min at 30 frames/second. Larval crawling speed was analyzed on the movie using a custom Matlab (MathWorks, Natick, MA) code: a larva was segmented from the background and larval centroid was determined every 30 frames (1 s). The distance that the larva traveled in 1 s was calculated from the coordinates of centroids. Larval speed (mm/sec) was calculated every 30 frames and the highest speed that the larva scored in 1 min was marked as peak locomotion speed. Average locomotion speed (mm/min) was calculated as total traveled distance per minute.

### Statistics

Statistical analysis was performed using GraphPad Prism (GraphPad Software, San Diego, CA) in conjunction with G*Power (University of Düsseldorf, Düsseldorf). Experimental data were analyzed using one-way ANOVA based on the previous studies^71^ without randomization and blinding. For multiple comparisons among relevant groups, Tukey or Dunnett’s post hoc test was used. Significance levels in the figures are represented as *p* < 0.05 (*), *p* < 0.01 (**), *p* < 0.001 (***), and *p* < 0.0001 (****). Error bars in the graphs represent standard errors of means. The statistics data are summarized in Supplementary Table S1.

### Data availability

All statistical data are deposited in Supplementary Table 1, which is available at the journal’s website. Other data sets including the confocal images, the genetic schemes, and the behavioral programs that were generated and/or analyzed during the current study are available from the corresponding author on reasonable request.

## Electronic supplementary material

Supplemental material
