# Supplementary material for: Genetic interaction of DISC1 and Neurexin in the development of fruit fly glutamatergic synapses
Source: NPJ Schizophr. 2017 Oct 27;3:39. doi: 10.1038/s41537-017-0040-6 (PMC5660244; doi:10.1038/s41537-017-0040-6)
Supplement: Supplementary file 1 — Supplemental material [file 41537_2017_40_MOESM1_ESM.pdf]

Supplementary Table 1. Summary of Statistical Analyses

Figure 2G. Total Bouton Area / NMJ

One-way ANOVA

F (DFn, DFd)	P value	Significance
F (5, 85) = 7.49	P<0.0001	****

Tukey's Multiple Comparisons Test

Comparisons	Significance	P value	q	DF
+/+ DISC1 (-) vs. +/+ DISC1 (+)	**	0.0021	5.589	85
+/+ DISC1 (-) vs. dnrx1/+ DISC1 (-)	ns	0.9853	0.9396	85
+/+ DISC1 (-) vs. dnrx1/+ DISC1 (+)	ns	0.3963	2.718	85
+/+ DISC1 (-) vs. dnrx1 RNAi DISC1 (-)	ns	0.7443	1.938	85
+/+ DISC1 (-) vs. dnrx1 RNAi DISC1 (+)	ns	0.9998	0.3898	85
+/+ DISC1 (+) vs. dnrx1/+ DISC1 (-)	***	0.0009	5.949	85
+/+ DISC1 (+) vs. dnrx1/+ DISC1 (+)	****	<0.0001	7.377	85
+/+ DISC1 (+) vs. dnrx1 RNAi DISC1 (-)	***	0.0004	6.253	85
+/+ DISC1 (+) vs. dnrx1 RNAi DISC1 (+)	*	0.0281	4.425	85
dnrx1/+ DISC1 (-) vs. dnrx1/+ DISC1 (+)	ns	0.8366	1.694	85
dnrx1/+ DISC1 (-) vs. dnrx1 RNAi DISC1 (-)	ns	0.9752	1.058	85
dnrx1/+ DISC1 (-) vs. dnrx1 RNAi DISC1 (+)	ns	0.9999	0.3328	85
dnrx1/+ DISC1 (+) vs. dnrx1 RNAi DISC1 (-)	ns	0.9993	0.4905	85
dnrx1/+ DISC1 (+) vs. dnrx1 RNAi DISC1 (+)	ns	0.8248	1.729	85
dnrx1 RNAi DISC1 (-) vs. dnrx1 RNAi DISC1 (+)	ns	0.9569	1.203	85

Figure 2H. Number of Boutons / NMJ

One-way ANOVA

F (DFn, DFd)	P value	Significance
F (5, 82) = 3.19	P=0.0111	*

Tukey's Multiple Comparisons Test

Comparisons	Significance	P value	q	DF
+/+ DISC1 (-) vs. +/+ DISC1 (+)	ns	0.9216	1.393	82
+/+ DISC1 (-) vs. dnrx1/+ DISC1 (-)	ns	0.0901	3.792	82
+/+ DISC1 (-) vs. dnrx1/+ DISC1 (+)	ns	0.0633	3.996	82
+/+ DISC1 (-) vs. dnrx1 RNAi DISC1 (-)	ns	0.9909	0.846	82
+/+ DISC1 (-) vs. dnrx1 RNAi DISC1 (+)	ns	0.9908	0.8467	82
+/+ DISC1 (+) vs. dnrx1/+ DISC1 (-)	ns	0.5231	2.432	82
+/+ DISC1 (+) vs. dnrx1/+ DISC1 (+)	ns	0.3835	2.749	82
+/+ DISC1 (+) vs. dnrx1 RNAi DISC1 (-)	ns	0.7419	1.944	82
+/+ DISC1 (+) vs. dnrx1 RNAi DISC1 (+)	ns	>0.9999	0.1928	82
dnrx1/+ DISC1 (-) vs. dnrx1/+ DISC1 (+)	ns	0.9993	0.4929	82
dnrx1/+ DISC1 (-) vs. dnrx1 RNAi DISC1 (-)	ns	0.0761	3.891	82
dnrx1/+ DISC1 (-) vs. dnrx1 RNAi DISC1 (+)	ns	0.688	2.07	82
dnrx1/+ DISC1 (+) vs. dnrx1 RNAi DISC1 (-)	ns	0.0523	4.103	82
dnrx1/+ DISC1 (+) vs. dnrx1 RNAi DISC1 (+)	ns	0.5487	2.376	82
dnrx1 RNAi DISC1 (-) vs. dnrx1 RNAi DISC1 (+)	ns	0.916	1.417	82

Figure 2I. Number of Branchpoints / NMJ

One-way ANOVA

F (DFn, DFd)	P value	Significance
F (5, 84) = 7.08	P<0.0001	****

Tukey's Multiple Comparisons Test

Comparisons	Significance	P value	q	DF
+/+ DISC1 (-) vs. +/+ DISC1 (+)	ns	0.1536	3.455	84
+/+ DISC1 (-) vs. dnrx1/+ DISC1 (-)	ns	0.9265	1.37	84
+/+ DISC1 (-) vs. dnrx1/+ DISC1 (+)	***	0.0009	5.956	84
+/+ DISC1 (-) vs. dnrx1 RNAi DISC1 (-)	ns	0.7223	1.991	84
+/+ DISC1 (-) vs. dnrx1 RNAi DISC1 (+)	****	<0.0001	6.963	84
+/+ DISC1 (+) vs. dnrx1/+ DISC1 (-)	ns	0.7915	1.819	84
+/+ DISC1 (+) vs. dnrx1/+ DISC1 (+)	ns	0.3481	2.836	84
+/+ DISC1 (+) vs. dnrx1 RNAi DISC1 (-)	ns	0.9937	0.7803	84
+/+ DISC1 (+) vs. dnrx1 RNAi DISC1 (+)	*	0.0355	4.307	84
dnrx1/+ DISC1 (-) vs. dnrx1/+ DISC1 (+)	*	0.0333	4.34	84
dnrx1/+ DISC1 (-) vs. dnrx1 RNAi DISC1 (-)	ns	0.9943	0.7641	84
dnrx1/+ DISC1 (-) vs. dnrx1 RNAi DISC1 (+)	**	0.0023	5.57	84
dnrx1/+ DISC1 (+) vs. dnrx1 RNAi DISC1 (-)	ns	0.2458	3.121	84
dnrx1/+ DISC1 (+) vs. dnrx1 RNAi DISC1 (+)	ns	0.8132	1.761	84
dnrx1 RNAi DISC1 (-) vs. dnrx1 RNAi DISC1 (+)	*	0.0276	4.435	84

Figure 2J. SYT / HRP

One-way ANOVA

F (DFn, DFd)	P value	Significance
F (5, 68) = 0.22	P=0.9550	ns

Tukey's Multiple Comparisons Test

Comparisons	Significance	P value	q	DF
+/+ DISC1 (-) vs. +/+ DISC1 (+)	ns	>0.9999	0.1499	68
+/+ DISC1 (-) vs. dnrx1/+ DISC1 (-)	ns	>0.9999	0.06829	68
+/+ DISC1 (-) vs. dnrx1/+ DISC1 (+)	ns	>0.9999	0.3044	68
+/+ DISC1 (-) vs. dnrx1 RNAi DISC1 (-)	ns	0.991	0.8425	68
+/+ DISC1 (-) vs. dnrx1 RNAi DISC1 (+)	ns	0.9989	0.5394	68
+/+ DISC1 (+) vs. dnrx1/+ DISC1 (-)	ns	>0.9999	0.2197	68
+/+ DISC1 (+) vs. dnrx1/+ DISC1 (+)	ns	0.9995	0.4542	68
+/+ DISC1 (+) vs. dnrx1 RNAi DISC1 (-)	ns	0.9974	0.6452	68
+/+ DISC1 (+) vs. dnrx1 RNAi DISC1 (+)	ns	0.9998	0.3644	68
dnrx1/+ DISC1 (-) vs. dnrx1/+ DISC1 (+)	ns	>0.9999	0.2397	68
dnrx1/+ DISC1 (-) vs. dnrx1 RNAi DISC1 (-)	ns	0.9847	0.9462	68
dnrx1/+ DISC1 (-) vs. dnrx1 RNAi DISC1 (+)	ns	0.9978	0.6266	68
dnrx1/+ DISC1 (+) vs. dnrx1 RNAi DISC1 (-)	ns	0.9466	1.265	68
dnrx1/+ DISC1 (+) vs. dnrx1 RNAi DISC1 (+)	ns	0.9875	0.9049	68
dnrx1 RNAi DISC1 (-) vs. dnrx1 RNAi DISC1 (+)	ns	>0.9999	0.2896	68

Figure 2K. HRP / NMJ

One-way ANOVA

F (DFn, DFd)	P value	Significance
F (5, 68) = 1.72	P=0.1423	ns

Tukey's Multiple Comparisons Test

Comparisons	Significance	P value	q	DF
+/+ DISC1 (-) vs. +/+ DISC1 (+)	ns	0.9998	0.3771	68
+/+ DISC1 (-) vs. dnrx1/+ DISC1 (-)	ns	0.5386	2.4	68
+/+ DISC1 (-) vs. dnrx1/+ DISC1 (+)	ns	0.2324	3.171	68
+/+ DISC1 (-) vs. dnrx1 RNAi DISC1 (-)	ns	0.3126	2.935	68
+/+ DISC1 (-) vs. dnrx1 RNAi DISC1 (+)	ns	0.4539	2.588	68
+/+ DISC1 (+) vs. dnrx1/+ DISC1 (-)	ns	0.7405	1.947	68
+/+ DISC1 (+) vs. dnrx1/+ DISC1 (+)	ns	0.4155	2.677	68
+/+ DISC1 (+) vs. dnrx1 RNAi DISC1 (-)	ns	0.5336	2.411	68
+/+ DISC1 (+) vs. dnrx1 RNAi DISC1 (+)	ns	0.6702	2.111	68
dnrx1/+ DISC1 (-) vs. dnrx1/+ DISC1 (+)	ns	0.9963	0.6967	68
dnrx1/+ DISC1 (-) vs. dnrx1 RNAi DISC1 (-)	ns	>0.9999	0.2846	68
dnrx1/+ DISC1 (-) vs. dnrx1 RNAi DISC1 (+)	ns	>0.9999	0.09772	68
dnrx1/+ DISC1 (+) vs. dnrx1 RNAi DISC1 (-)	ns	0.9993	0.4849	68
dnrx1/+ DISC1 (+) vs. dnrx1 RNAi DISC1 (+)	ns	0.9978	0.6256	68
dnrx1 RNAi DISC1 (-) vs. dnrx1 RNAi DISC1 (+)	ns	>0.9999	0.1893	68

Figure 2L. DNRX1 / HRP

One-way ANOVA

F (DFn, DFd)	P value	Significance
F (2, 47) = 22.89	P<0.0001	****

Tukey's Multiple Comparisons Test

Comparisons	Significance	P value	q	DF
+/+ vs. dnrx1/+	****	<0.0001	7.974	47
+/+ vs. dnrx1 RNAi	****	<0.0001	8.403	47
dnrx1/+ vs. dnrx1 RNAi	ns	0.7833	0.9442	47

Figure 3E. BRP / HRP

One-way ANOVA

F (DFn, DFd)	P value	Significance
F (3, 87) = 32.73	P<0.0001	****

Tukey's Multiple Comparisons Test

Comparisons	Significance	P value	q	DF
+/+ DISC1 (-) vs. +/+ DISC1 (+)	*	0.02	4.195	87
+/+ DISC1 (-) vs. dnrx1/+ DISC1 (-)	****	<0.0001	8.07	87
+/+ DISC1 (-) vs. dnrx1/+ DISC1 (+)	****	<0.0001	13.51	87
+/+ DISC1 (+) vs. dnrx1/+ DISC1 (-)	ns	0.0622	3.579	87
+/+ DISC1 (+) vs. dnrx1/+ DISC1 (+)	****	<0.0001	9.109	87
dnrx1/+ DISC1 (-) vs. dnrx1/+ DISC1 (+)	***	0.0005	5.86	87

Figure 3F. Active Zone Density

One-way ANOVA

F (DFn, DFd)	P value	Significance
F (3, 96) = 7.22	P=0.0002	***

Tukey's Multiple Comparisons Test

Comparisons	Significance	P value	q	DF
+/+ DISC1 (-) vs. +/+ DISC1 (+)	**	0.0049	4.849	96
+/+ DISC1 (-) vs. dnrx1/+ DISC1 (-)	***	0.0003	6.031	96
+/+ DISC1 (-) vs. dnrx1/+ DISC1 (+)	ns	0.2714	2.571	96
+/+ DISC1 (+) vs. dnrx1/+ DISC1 (-)	ns	0.8284	1.208	96
+/+ DISC1 (+) vs. dnrx1/+ DISC1 (+)	ns	0.6615	1.622	96
dnrx1/+ DISC1 (-) vs. dnrx1/+ DISC1 (+)	ns	0.2355	2.685	96

Figure 3O. DGLURIIA / HRP

One-way ANOVA

F (DFn, DFd)	P value	Significance
F (3, 83) = 96.4	P<0.0001	****

Tukey's Multiple Comparisons Test

Comparisons	Significance	P value	q	DF
+/+ DISC1 (-) vs. +/+ DISC1 (+)	*	0.0216	4.16	83
+/+ DISC1 (-) vs. dnrx1/+ DISC1 (-)	****	<0.0001	17.65	83
+/+ DISC1 (-) vs. dnrx1/+ DISC1 (+)	****	<0.0001	19.51	83
+/+ DISC1 (+) vs. dnrx1/+ DISC1 (-)	****	<0.0001	13.99	83
+/+ DISC1 (+) vs. dnrx1/+ DISC1 (+)	****	<0.0001	16.07	83
dnrx1/+ DISC1 (-) vs. dnrx1/+ DISC1 (+)	ns	0.2194	2.744	83

Figure 3P. DLG / HRP

One-way ANOVA

F (DFn, DFd)	P value	Significance
F (3, 77) = 20.8	P<0.0001	****

Tukey's Multiple Comparisons Test

Comparisons	Significance	P value	q	DF
+/+ DISC1 (-) vs. +/+ DISC1 (+)	ns	0.9911	0.4161	77
+/+ DISC1 (-) vs. dnrx1/+ DISC1 (-)	ns	0.1419	3.064	77
+/+ DISC1 (-) vs. dnrx1/+ DISC1 (+)	****	<0.0001	10	77
+/+ DISC1 (+) vs. dnrx1/+ DISC1 (-)	ns	0.2377	2.683	77
+/+ DISC1 (+) vs. dnrx1/+ DISC1 (+)	****	<0.0001	9.713	77
dnrx1/+ DISC1 (-) vs. dnrx1/+ DISC1 (+)	****	<0.0001	7.104	77

Figure 4E. Central / Peripheral Signal Ratio

One-way ANOVA

F (DFn, DFd)	P value	Significance
F (3, 122) = 45.4	P<0.0001	****

Tukey's Multiple Comparisons Test

Comparisons	Significance	P value	q	DF
+/+ DISC1 (-) vs. +/+ DISC1 (+)	ns	0.7127	1.503	122
+/+ DISC1 (-) vs. dnrx1/+ DISC1 (-)	ns	0.9939	0.3657	122
+/+ DISC1 (-) vs. dnrx1/+ DISC1 (+)	****	<0.0001	12.35	122
+/+ DISC1 (+) vs. dnrx1/+ DISC1 (-)	ns	0.5939	1.773	122
+/+ DISC1 (+) vs. dnrx1/+ DISC1 (+)	****	<0.0001	13.44	122
dnrx1/+ DISC1 (-) vs. dnrx1/+ DISC1 (+)	****	<0.0001	11.04	122

Figure 5G. DNRX1 / HRP

One-way ANOVA

F (DFn, DFd)	P value	Significance
F (5, 72) = 20.63	P<0.0001	****

Tukey's Multiple Comparisons Test

Comparisons	Significance	P value	q	DF
tubP DISC1 (-) vs. tubP DISC1 (+)	**	0.009	5.001	72
elav DISC1 (-) vs. elav DISC1 (+)	*	0.0331	4.362	72
C57 DISC1 (-) vs. C57 DISC1 (+)	ns	0.6596	2.134	72

Figure 5H. HRP / NMJ

One-way ANOVA

F (DFn, DFd)	P value	Significance
F (5, 72) = 17.6	P<0.0001	****

Tukey's Multiple Comparisons Test

Comparisons	Significance	P value	q	DF
tubP DISC1 (-) vs. tubP DISC1 (+)	ns	0.5606	2.351	72
elav DISC1 (-) vs. elav DISC1 (+)	ns	0.6613	2.13	72
C57 DISC1 (-) vs. C57 DISC1 (+)	ns	0.2191	3.213	72

Figure 6H. DNRX1 / HRP

One-way ANOVA

F (DFn, DFd)	P value	Significance
F (5, 87) = 100.6	P<0.0001	****

Dunnett's Multiple Comparisons Test

Comparisons	Significance	P value	q	DF
FL (1-854) vs. Control	****	0.0001	5.372	87
FL (1-854) vs. 1-597	****	0.0001	10.52	87
FL (1-854) vs. 1-402	ns	0.1108	2.222	87
FL (1-854) vs. mtNLS1	****	0.0001	9.378	87
FL (1-854) vs. 291-854	ns	0.0688	2.429	87

Dunnett's Multiple Comparisons Test

Comparisons	Significance	P value	q	DF
Control vs. FL (1-854)	****	0.0001	5.372	87
Control vs. 1-597	****	0.0001	17.89	87
Control vs. 1-402	****	0.0001	9.101	87
Control vs. mtNLS1	****	0.0001	16.16	87
Control vs. 291-854	*	0.0108	3.122	87

Figure 6I. HRP / NMJ

One-way ANOVA

F (DFn, DFd)	P value	Significance
F (5, 87) = 1.79	P=0.1224	ns

Dunnett's Multiple Comparisons Test

Comparisons	Significance	P value	q	DF
FL (1-854) vs. Control	ns	0.9631	0.6001	87
FL (1-854) vs. 1-597	ns	0.36	1.608	87
FL (1-854) vs. 1-402	ns	0.0738	2.4	87
FL (1-854) vs. mtNLS1	ns	0.2194	1.889	87
FL (1-854) vs. 291-854	ns	0.9252	0.719	87

Dunnett's Multiple Comparisons Test

Comparisons	Significance	P value	q	DF
Control vs. FL (1-854)	ns	0.9631	0.6001	87
Control vs. 1-597	ns	0.7181	1.082	87
Control vs. 1-402	ns	0.1961	1.947	87
Control vs. mtNLS1	ns	0.4841	1.412	87
Control vs. 291-854	ns	0.9998	0.1401	87

Figure S1A. Average Locomotor Speed

One-way ANOVA

F (DFn, DFd)	P value	Significance
F (3, 75) = 5.798	P=0.0013	**

Tukey's Multiple Comparisons Test

Comparisons	Significance	P value	q	DF
+/+ DISC1 (-) vs. +/+ DISC1 (+)	ns	0.194	2.841	75
+/+ DISC1 (-) vs. dnrx1/+ DISC1 (-)	ns	>0.9999	0.05913	75
+/+ DISC1 (-) vs. dnrx1/+ DISC1 (+)	**	0.0037	5.018	75
+/+ DISC1 (+) vs. dnrx1/+ DISC1 (-)	ns	0.21	2.781	75
+/+ DISC1 (+) vs. dnrx1/+ DISC1 (+)	ns	0.3654	2.313	75
dnrx1/+ DISC1 (-) vs. dnrx1/+ DISC1 (+)	**	0.0042	4.96	75

Figure S1B. Peak Locomotor Speed

One-way ANOVA

F (DFn, DFd)	P value	Significance
F (3, 75) = 8.879	P<0.0001	****

Tukey's Multiple Comparisons Test

Comparisons	Significance	P value	q	DF
+/+ DISC1 (-) vs. +/+ DISC1 (+)	ns	0.1031	3.278	75
+/+ DISC1 (-) vs. dnrx1/+ DISC1 (-)	ns	0.924	0.8828	75
+/+ DISC1 (-) vs. dnrx1/+ DISC1 (+)	***	0.0009	5.607	75
+/+ DISC1 (+) vs. dnrx1/+ DISC1 (-)	*	0.0216	4.171	75
+/+ DISC1 (+) vs. dnrx1/+ DISC1 (+)	ns	0.303	2.482	75
dnrx1/+ DISC1 (-) vs. dnrx1/+ DISC1 (+)	***	0.0001	6.466	75

Figure S2E. Average Cell Size

One-way ANOVA

F (DFn, DFd)	P value	Significance
F (3, 189) = 2.04	P=0.1101	ns

Tukey's Multiple Comparisons Test

Comparisons	Significance	P value	q	DF
+/+ DISC1 (-) vs. +/+ DISC1 (+)	ns	0.9938	0.3687	189
+/+ DISC1 (-) vs. dnrx1/+ DISC1 (-)	ns	0.9856	0.4904	189
+/+ DISC1 (-) vs. dnrx1/+ DISC1 (+)	ns	0.1554	2.976	189
+/+ DISC1 (+) vs. dnrx1/+ DISC1 (-)	ns	0.9395	0.8129	189
+/+ DISC1 (+) vs. dnrx1/+ DISC1 (+)	ns	0.1291	3.101	189
dnrx1/+ DISC1 (-) vs. dnrx1/+ DISC1 (+)	ns	0.3003	2.477	189

Legends to the Supplementary Figures.

Figure S1. Quantification of larval locomotor activity.

(a) Average locomotion speed. (b) Peak locomotion speed. One-way ANOVA followed by Tukey's post-hoc test for multiple comparisons. $^{**}p < 0.01$, $^{***}p < 0.001$. Data are means ± SEM. Number of each sample is indicated at the bottom of the bar. The statistical data are listed in Supplementary Table 1.

Figure S2. Quantification of cell size.

(a-d) Confocal images of cell bodies in the larval ventral nerve cord immune-stained with anti-HRP (green) and TOPRO3 (magenta). Scale bar, 20µm. (e) Quantification of cell size. The area of each cell was measured by Image J based on confocal optical sections. Data are means ± SEM. One-way ANOVA. Number of sample is indicated at the bottom of the bar. The statistical values are listed in Supplementary Table 1.

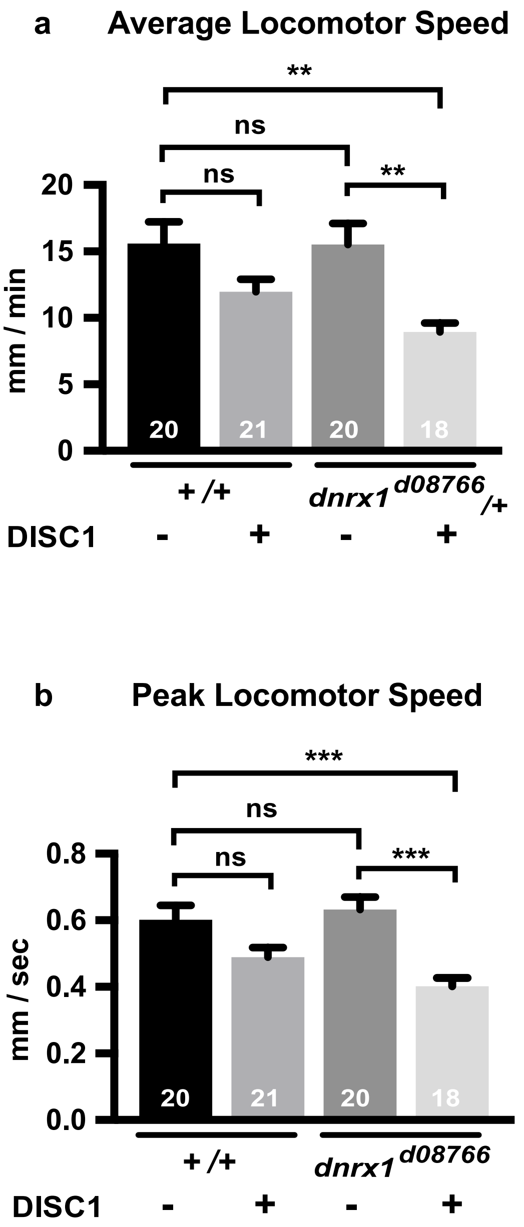
a
Average Locomotor Speed
**
ns
ns
**
20
15
10
5
0
mm / min
20
21
20
18
+ /+
dnrx1 d08766/+
DISC1
-
+
-
+
b
Peak Locomotor Speed
***
ns
ns
***
0.8
0.6
0.4
0.2
0.0
mm / sec
20
21
20
18
+ /+
dnrx1 d08766
DISC1
-
+
-
+

Figure S1

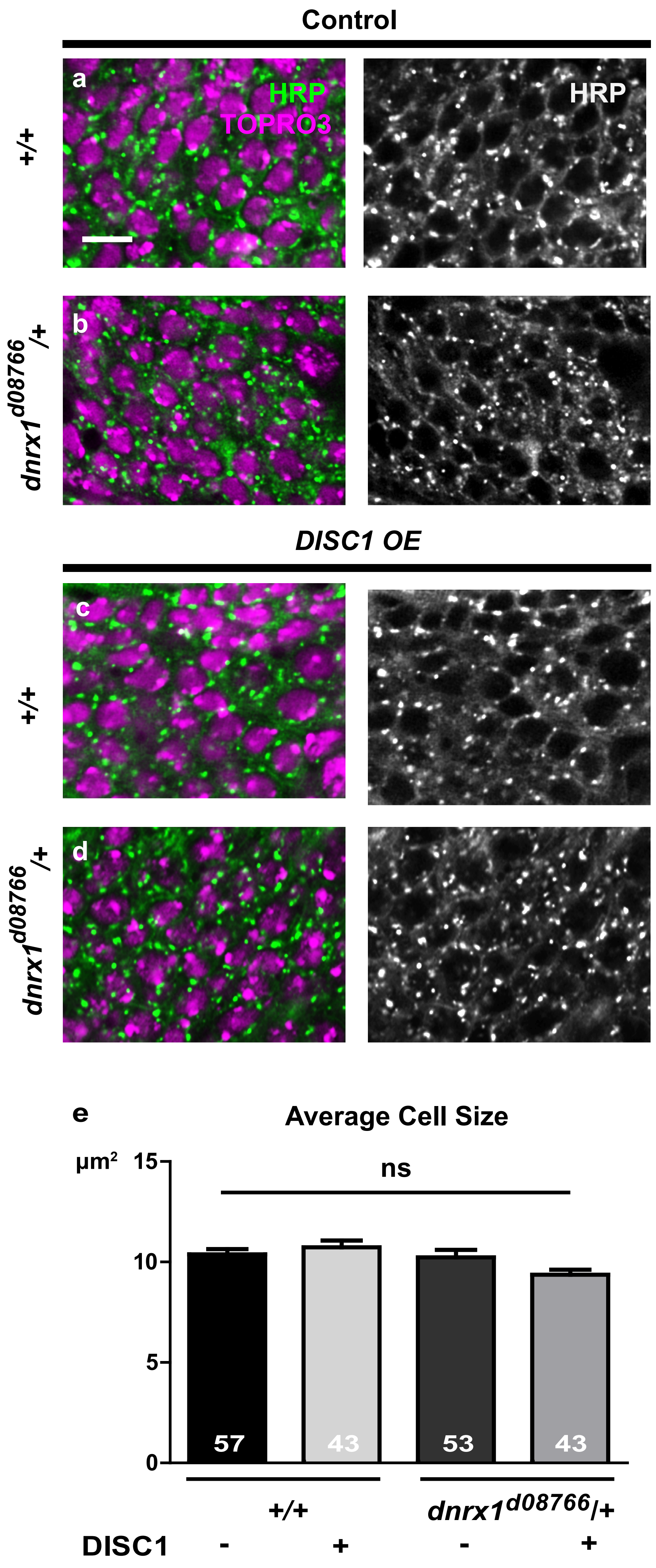

Control
a
HRP
TOPRO3
HRP
+/+
b
dnrx1d08766/+
DISC1 OE
c
+/+
d
dnrx1d08766/+
e
Average Cell Size
µm²
15
10
5
0
ns
57
43
53
43
+/+
dnrx1d08766/+
DISC1
-
+
-
+

Figure S2
